# Elucidating the Roles of Amorphous Alumina Overcoat in Palladium-Catalyzed Selective Hydrogenation

**DOI:** 10.1021/acsami.2c02132

**Published:** 2022-05-18

**Authors:** Divakar R. Aireddy, Haoran Yu, David A. Cullen, Kunlun Ding

**Affiliations:** † Department of Chemical Engineering, Louisiana State University, Baton Rouge, Louisiana 70803, United States; ‡ Center for Nanophase Materials Sciences, Oak Ridge National Laboratory, Oak Ridge, Tennessee 37831, United States

**Keywords:** atomic layer deposition, amorphous overcoat, heterolytic hydrogen dissociation, hydrogen spillover, selective hydrogenation

## Abstract

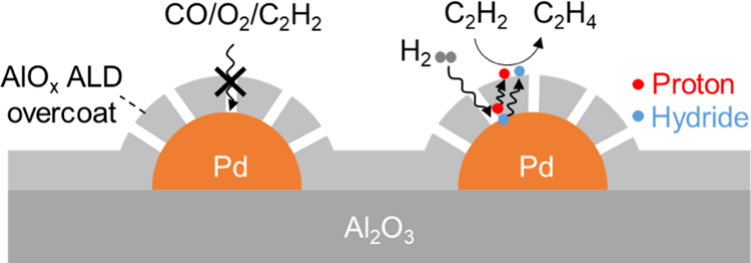

Amorphous alumina overcoats generated by atomic layer deposition (ALD) have been shown to improve the selectivity and durability of supported metal catalysts in many reactions. Several mechanisms have been proposed to explain the enhanced catalytic performance, but the accessibilities of reactants through the amorphous overcoats remain elusive, which is crucial for understanding reaction mechanisms. Here, we show that an AlO*_x_* ALD overcoat is able to improve the alkene product selectivity of a supported Pd catalyst in acetylene (C_2_H_2_) hydrogenation. We further demonstrate that the AlO*_x_* ALD overcoat blocks the access of C_2_H_2_ (kinetic diameter of 0.33 nm), O_2_ (0.35 nm), and CO (0.38 nm) but allows H_2_ (0.29 nm) to access Pd surfaces. A H–D exchange experiment suggests that H_2_ might dissociate heterolytically at the Pd–AlO*_x_* interface. These findings are in favor of a hydrogen spillover mechanism.

## Introduction

Atomic layer deposition (ALD) has been demonstrated as a powerful tool for encapsulating metal nanoparticles (NPs) to improve their catalytic performance in heterogeneous catalysis.^1−6^ Superior selectivity and durability have been observed in many reactions, including alkane dehydrogenation,^7^ oxidative dehydrogenation,^8,9^ selective hydrogenation of alkynes^10,11^/dienes^12^ and unsaturated aldehydes,^13,14^ methanol decomposition,^15^ CO oxidation,^16,17^ and methane combustion.^18^ The improvement in catalytic performance by an ALD overcoat has been mostly attributed to the following factors: (i) physical confinement of metal NPs, which restricts the coalescence/sintering;^7,8^ (ii) selective blockage of certain crystal facets or low-coordinated sites;^7,8,19^ (iii) confinement effect of diffusion and adsorption due to the microporous nature of the oxide overcoat;^12,19^ (iv) size reduction of adsorption site ensembles;^12,18^ and (v) unique active site structures at inverse metal–oxide interfaces.^14,16,17^

A hydrogen spillover mechanism has also been introduced to explain the enhanced hydrogenation selectivity by ALD overcoating.^13,20,21^ The hydrogen spillover effect in heterogeneous catalysis has been studied since the 1960s.^22−25^ Most studies focused on spillover from metals to reducible metal oxides^22−25^ and metals to metals.^26−28^ For hydrogen spillover onto a reducible support, H_2_ evolves into two protons by donating two electrons to the conduction band of metal oxides and thereby partially reduces the oxides (TiO_2_,^29,30^ WO_3_,^22−24^ MoO_3_,^31^ etc.). In contrast, for hydrogen spillover between metals, H_2_ dissociates into two nearly neutral hydrogen atoms.^26−28^

The occurrence of hydrogen spillover from metals to irreducible supports (e.g., Al_2_O_3_,^32^ MgO,^25^ SiO_2_^33,34^) has been controversial. Karim et al. studied hydrogen spillover by separating Pt and iron oxide particles with precisely controlled distances on TiO_2_ and Al_2_O_3_ surfaces via nanofabrication.^32^ In Karim’s work, H_2_ dissociates on Pt and spills over the support and reduces iron oxide particles. By measuring the reduction degree of iron oxide, they demonstrated that hydrogen spillover on Al_2_O_3_ was slower by 10 orders of magnitude compared to that on TiO_2_ and the former was restricted to a very short distance from Pt particles (<15 nm). DFT calculations reveal that the fast hydrogen spillover on TiO_2_ takes place via coupled proton–electron transfer mechanism. In contrast, the hydrogen spillover on Al_2_O_3_ is mediated by O–H^δ+^/Al–H^δ−^ pairs.^32^ One important implication from this study is that hydrogen spillover onto irreducible oxide, though difficult, may still occur within very short distances (a few nanometers) from metal particles. Given the fact that the thicknesses of ALD overcoat layers in catalytic studies mostly fall in the range of 1–5 nm, is it possible that hydrogen spillover plays appreciable roles in hydrogenation reactions catalyzed by AlO*_x_*-overcoated metal catalysts?

Another information that is crucial for understanding the influence of ALD overcoats in heterogeneous catalysis is the accessibility of catalytic sites by various reactant molecules. Lu et al. discovered that an AlO*_x_* ALD overcoat on Pd NPs cracked at 700 °C and enabled the oxidative dehydrogenation of propane.^8^ However, the crystallization of the AlO*_x_* ALD overcoat does not take place until the annealing temperature is greater than 800 °C.^35,36^ As-synthesized AlO*_x_* ALD overcoats have been known to exhibit an amorphous structure with a higher oxygen content than that of Al_2_O_3_.^37^ George et al. proposed that the structure of the as-synthesized AlO*_x_* ALD overcoat was close to amorphous boehmite, i.e., hydroxylated alumina.^38^ Despite these structural characterizations, the diffusion/permeation behavior through an amorphous AlO*_x_* ALD overcoat has not been well understood. Infrared (IR) spectroscopy with CO as a probe molecule has been commonly used to evaluate the accessibility of metal sites underneath the ALD overcoat.^8,12^ What about the accessibilities of other molecules that are smaller than CO (kinetic diameter of 0.38 nm)?

To address these fundamental questions, we fabricated an AlO*_x_* overcoat with a thickness of 3–4 nm on supported Pd NPs. The overcoated Pd catalysts exhibited improved alkene product selectivity in competitive acetylene hydrogenation. We further investigated the accessibility of overcoated Pd sites by several small molecules including H_2_ (kinetic diameter of 0.29 nm), C_2_H_2_ (0.33 nm), O_2_ (0.35 nm), and CO (0.38 nm). The H_2_ accessibility was probed by H–D exchange between H_2_ and D_2_. The accessibility of C_2_H_2_ and O_2_ was probed by X-ray diffraction (XRD) based on the formation of PdC*_x_* and PdO phases. CO IR spectroscopy was adopted to study the accessibility of CO. We show that the AlO*_x_* overcoat layer allows the access of Pd by H_2_ but not C_2_H_2_, O_2_, or CO. Furthermore, the activation energy of H–D exchange between H_2_ and D_2_ suggests that the dihydrogen dissociation might undergo a heterolytic pathway. These findings suggest a hydrogen spillover mechanism for acetylene hydrogenation.

## Results and Discussion

**Figure 1:**
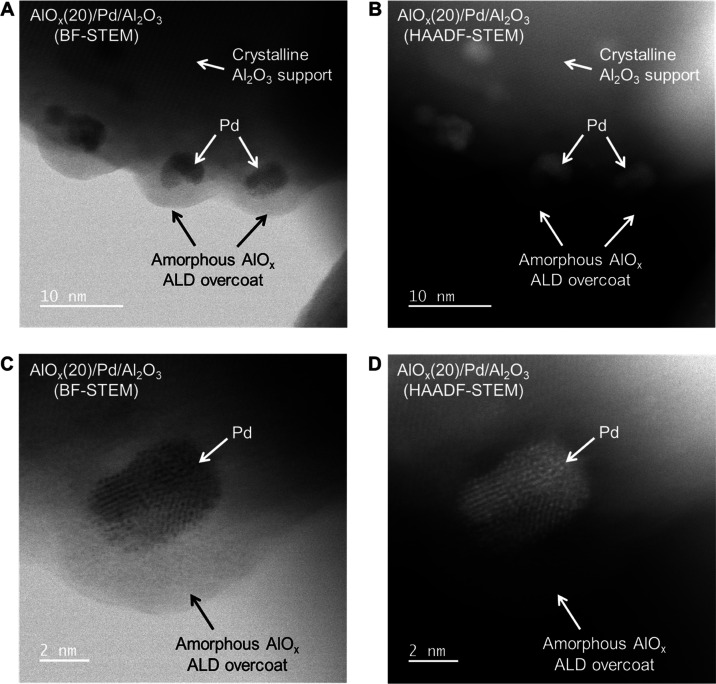
BF-STEM (A, C) and HAADF-STEM (B, D) images of AlO*_x_*(20)/Pd/Al_2_O_3_.

Colloidal Pd NPs were synthesized using polyethyleneimine (PEI) as a capping agent. The PEI-Pd NPs were adsorbed onto an Al_2_O_3_ support by our previously developed “antisolvent-induced adsorption” method.^39^ In this process, colloidal PEI-Pd NPs were gradually destabilized by the addition of a poor solvent, acetone, and completely adsorbed onto the support. Afterward, the sample was dried and then calcined to remove PEI. More details are provided in the Experimental Section. The transmission electron microscopy (TEM) images in Figures S1 and S2 showed that the calcined Pd/Al_2_O_3_ gave slightly bigger particle sizes of Pd NPs (3–4 nm) compared to those of as-synthesized PEI-Pd NPs (2–3 nm). Twenty cycles of AlO*_x_* ALD were carried out to overcoat Pd/Al_2_O_3_, denoted as AlO*_x_*(20)/Pd/Al_2_O_3_. The size of Pd NPs slightly increased after ALD (Figure S3). This is likely caused by sintering upon exposure to the ALD precursor, trimethylaluminum, which is a strong reducing agent. Aberration-corrected bright-field scanning transmission electron microscopy (BF-STEM) and high-angle annular dark-field (HAADF) STEM images in Figure 1 show the light-contrast amorphous AlO*_x_* shell with a thickness of 3–4 nm on Pd NPs. The lattice fringes of the crystalline Al_2_O_3_ support can also be clearly revealed and distinguished from the amorphous AlO*_x_* ALD overcoat (Figure 1A). No Pd species (bright spots in HAADF images) were detected outside the AlO*_x_* shell (Figure 1D). However, it is very challenging to visualize the “pores” in the amorphous AlO*_x_* materials given the extremely small pore size even if they exist. Understanding the structural features of amorphous AlO*_x_* ALD materials has been an active topic in ALD studies, as shown in some recent publications.^37,38^

**Figure 2:**
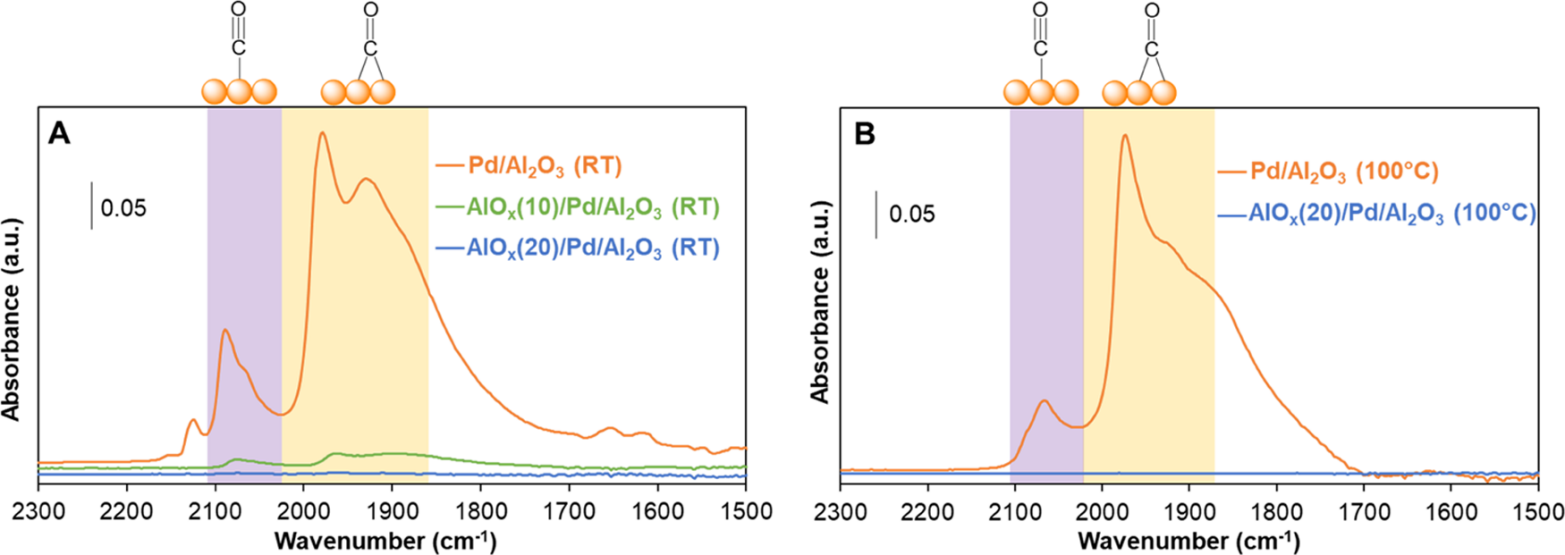
IR spectra of CO molecules adsorbed on uncoated and overcoated Pd/Al_2_O_3_ at different temperatures: (A) room temperature (RT) and (B) 100 °C.

IR spectroscopy with CO (0.38 nm) as a probe molecule was used to study the accessibility of Pd NPs before and after AlO*_x_* overcoating (Figure 2). Uncoated Pd/Al_2_O_3_ (Figure 2A) shows two strong bands at 1965 and 1915 cm^–1^ (bridge CO at twofold sites) and three weak bands at 2080, 2090, and 2110 cm^–1^ (linear CO at atop sites). The CO band intensity significantly reduced after 10 cycles of AlO*_x_* ALD (Figure 2A) and completely diminished after 20 cycles of AlO*_x_* ALD (Figure 2A), indicating that a 20-cycle AlO*_x_* ALD overcoat is able to completely encapsulate Pd NPs and prevent the access of CO. Similar IR results were also observed in CO adsorption at 100 °C (Figure 2B), revealing that the CO molecule cannot diffuse through the 20-cycle AlO*_x_* ALD overcoat even at elevated temperatures, which were used for evaluating the catalytic performance of these catalysts in acetylene hydrogenation.

**Figure 3:**
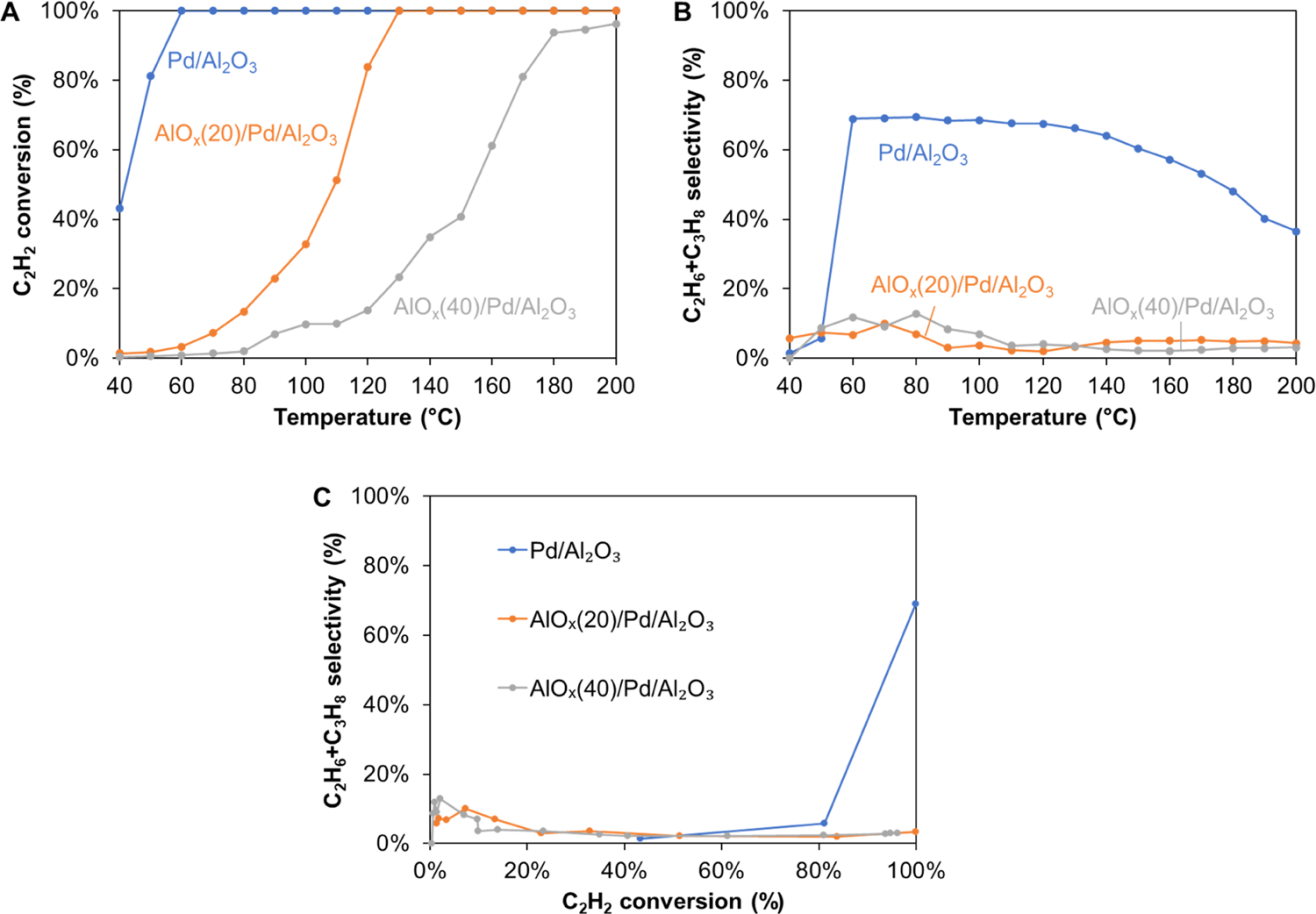
Catalytic performance of uncoated and overcoated Pd/Al_2_O_3_ in acetylene hydrogenation. (A) Acetylene conversion and (B) alkane selectivity as a function of temperature. (C) Alkane selectivity as a function of acetylene conversion.

The catalytic performance of the uncoated and overcoated catalysts was evaluated in acetylene hydrogenation under a competitive condition (in the presence of a large excess of propylene) (Figures 3 and S4–S6). Propylene was selected instead of ethylene to avoid the interference of product analysis since ethylene is also the major product of acetylene hydrogenation. Figure 3 shows that the C_2_H_2_ conversion reached 100% at 60 °C on uncoated Pd/Al_2_O_3_ with nearly 70% selectivity toward overhydrogenated products, alkanes (ethane and propane), confirming the high activity but poor selectivity of the unmodified Pd metal in alkyne hydrogenation. In contrast, AlO*_x_*(20)/Pd/Al_2_O_3_ showed lower activity but dramatically suppressed alkane selectivity at 100% C_2_H_2_ conversion (∼4% selectivity toward alkanes at 130 °C). The catalytic performance is comparable with those of the best catalysts reported in the literature.^40,41^ The C_2_H_2_ conversion on AlO*_x_*(40)/Pd/Al_2_O_3_ drastically decreased compared to that on AlO*_x_*(20)/Pd/Al_2_O_3_ (Figure 3), indicating that the catalytic activity is dependent on the thickness of the AlO*_x_* overcoat.

Since the uncoated Pd catalysts are much more active than the coated Pd, it is difficult to reach similar conversion vs *T* curves by simply adjusting space velocities. To overcome this issue, we decreased the Pd loading of uncoated catalysts from 2 to 0.25 and 0.1 wt % using an identical colloidal Pd NP adsorption protocol. The uncoated catalysts with lower Pd loadings showed lower alkane selectivity because of the decrease in the number of Pd sites (Figure S7). No significant difference in selectivity was observed between uncoated and coated Pd catalysts below 100% C_2_H_2_ conversion. However, after the C_2_H_2_ conversion reached 100%, the alkane selectivity over uncoated catalysts started to increase and became twice that on coated Pd catalysts. In other words, the product selectivity on coated Pd catalysts is less sensitive to C_2_H_2_ conversion compared to that on uncoated catalysts.

We further tested the catalytic performance of the AlO*_x_*(20)/Pd/Al_2_O_3_ sample after 600 °C calcination. The activity showed very limited increase with no noticeable change in product selectivity, as shown in Figure S8. In contrast, a more significant change in activity and selectivity was observed on the 700 °C calcined AlO*_x_*(20)/Pd/Al_2_O_3_ sample. CO IR shows much stronger CO peaks on the 700 °C calcined AlO*_x_*(20)/Pd/Al_2_O_3_ sample compared to those on the 600 °C calcined one, indicating that the AlO*_x_* ALD overcoat on AlO*_x_*(20)/Pd/Al_2_O_3_ might be broken at 700 °C (Figure S9).

It is worth noting that the improved alkene selectivity was reported on the AlO*_x_*-overcoated Pd catalyst in butadiene hydrogenation.^12^ The improvement was attributed to the confined adsorption geometry of butadiene on the AlO*_x_*-overcoated Pd surface. Since the kinetic diameter of butadiene (0.43 nm) is even greater than that of CO (0.38 nm), it remains questionable whether butadiene is able to diffuse through the AlO*_x_* ALD overcoat and access Pd surfaces.

**Figure 4:**
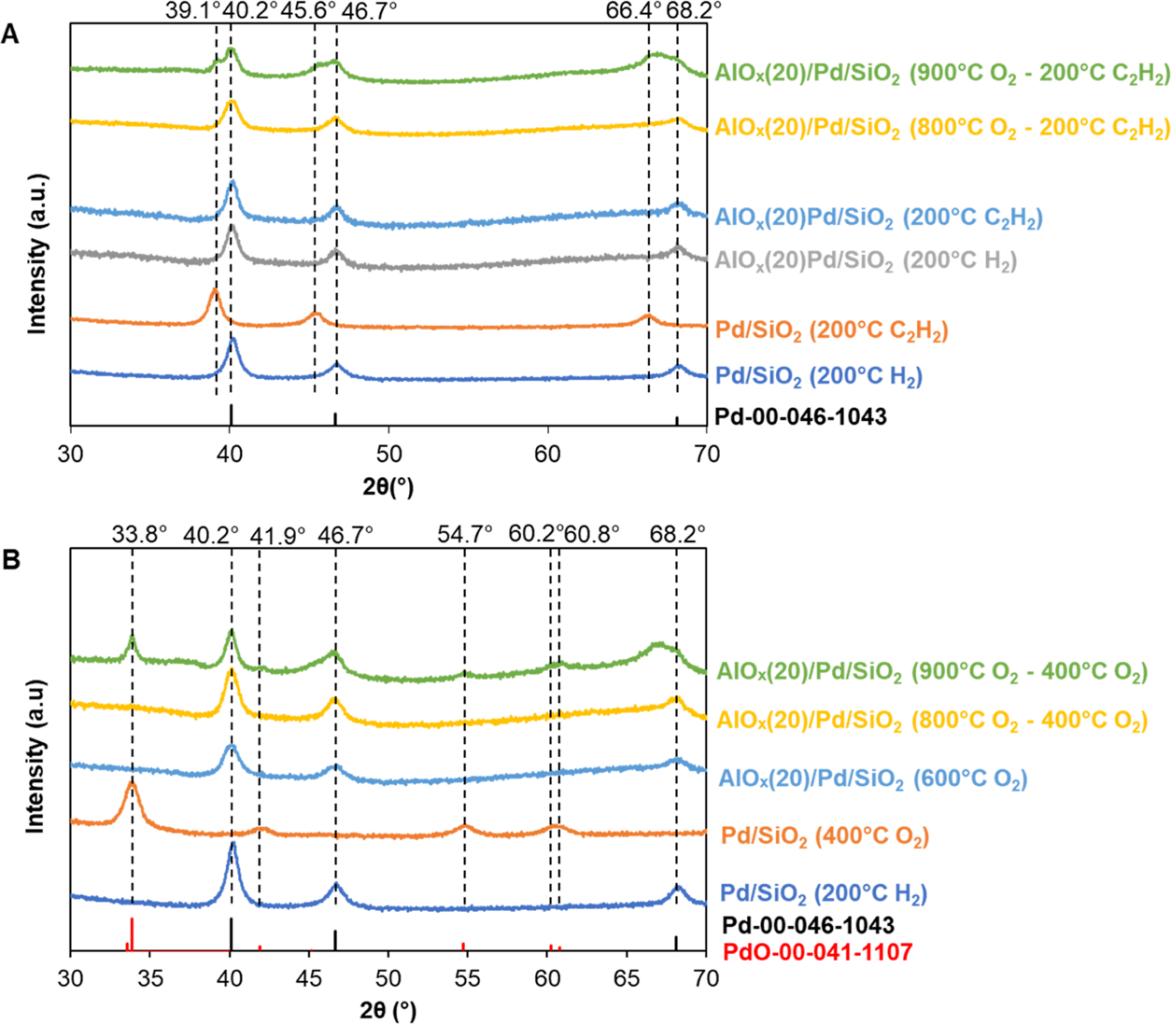
XRD patterns of coated and uncoated Pd/SiO_2_ after different treatments: (A) carburization and (B) calcination.

Next, we studied the accessibility of C_2_H_2_ molecules (0.33 nm) to Pd NPs. It has been reported that Pd would quickly convert to PdC*_x_* upon exposure to C_2_H_2_.^42−44^ The interstitial carbon atoms in PdC*_x_* result in a slight lattice expansion, which can be characterized by the XRD peak shift to lower angles. Therefore, XRD can be employed to probe the accessibility of C_2_H_2_ to Pd NPs. However, the small Pd NP size leads to peak broadening of XRD patterns. The strong XRD signals from the crystalline Al_2_O_3_ support further interfere with the data analysis. To address these issues, we synthesized Pd NPs with a size of ∼13 nm (Figure S10) and deposited them onto an amorphous fumed SiO_2_ support for XRD studies. CO IR characterizations of the uncoated Pd/SiO_2_ and overcoated AlO*_x_*(20)/Pd/SiO_2_ catalysts confirmed the absence of CO bands (Figure S11) upon ALD overcoating. Unlike the crystalline Al_2_O_3_ support, the fumed SiO_2_ exhibits a broad XRD peak below 30° scattering angle, which does not interfere with the XRD signals associated with Pd species. The XRD pattern of the reduced Pd/SiO_2_ showed a sharp peak at 40.2° (Figure 4A), which belongs to the metallic Pd phase.^18,42−45^ After 30 min of exposure to the C_2_H_2_ atmosphere at 200 °C, Pd was completely carburized and converted to the PdC*_x_* phase, as suggested by the shift of the XRD peak to a lower scattering angle (39.1°).^42−44^ The XRD pattern of overcoated AlO*_x_*(20)/Pd/SiO_2_ showed identical peaks of reduced Pd/SiO_2_. Interestingly, no peak shift was observed on AlO*_x_*(20)/Pd/SiO_2_ upon 30 min of exposure to C_2_H_2_ at 200 °C, indicating that the carburization of Pd was inhibited by the AlO*_x_* overcoat. In other words, the access of C_2_H_2_ to Pd NPs was effectively blocked by the AlO*_x_* overcoat.

Similarly, the conversion of the Pd to PdO phase upon calcination was used to probe the accessibility of O_2_ to Pd NPs through the AlO*_x_* overcoat (Figure 4B). Oxygen has a kinetic diameter of 0.35 nm, which is between C_2_H_2_ (0.33 nm) and CO (0.38 nm). In principle, O_2_ should not be able diffuse through the AlO*_x_* overcoat. This was confirmed by the absence of PdO peaks for overcoated AlO*_x_*(20)/Pd/SiO_2_ upon calcination at 600 °C, whereas the uncoated Pd/SiO_2_ converted to PdO/SiO_2_ upon calcination at 400 °C (Figure 4B).

To understand the thermal stability of the AlO*_x_* ALD overcoat, we further calcined the AlO*_x_*(20)/Pd/SiO_2_ sample at 800 and 900 °C in air. CO IR spectra show no significant CO adsorption at room temperature after 2 h of calcination at 800 °C. Noticeable CO peaks were observed after 900 °C calcination (Figure S11). After calcination at 800 and 900 °C, the samples were further calcined at 400 °C for 4 h before XRD analysis since PdO is known to decompose above 800 °C. Pd partially converted to PdO after 900 °C (2 h)–400 °C (4 h) calcination. However, the 800 °C (2 h)–400 °C (4 h) calcination sample only showed the Pd phase (Figure 4B). Similarly, we also observed the partial conversion of Pd to PdC*_x_* after 900 °C calcination and 200 °C carburization. No PdC*_x_* formation was detected for the 800 °C calcined and 200 °C carburized samples (Figure 4A). These results suggest that the AlO*_x_* ALD overcoat on AlO*_x_*(20)/Pd/SiO_2_ is thermally stable up to 800 °C. This behavior is somehow different from some literature studies on the thermal stability of the AlO*_x_* ALD overcoat where crystalline Al_2_O_3_ was used as a catalyst support, which showed that the amorphous AlO*_x_* ALD overcoat cracked below 700 °C.^8,38^ We suspect that the crystal epitaxy between the crystalline Al_2_O_3_ and amorphous AlO*_x_* might cause this difference. More research is needed to elucidate this behavior.

Interestingly, TEM images and size histograms clearly showed the sintering of Pd NPs upon 900 °C calcination regardless of the preservation in continuity of the AlO*_x_* ALD overcoat (Figure S12). It seems that Pd NPs are able to migrate under the AlO*_x_* ALD overcoat and fuse into larger NPs (as large as 25 nm), leaving behind empty pockets (Figure S12). The large Pd NPs are still covered by the AlO*_x_* ALD overcoat. This suggests that the AlO*_x_* ALD overcoat is flexible enough to accommodate the rather large volumetric expansion of Pd during agglomeration and fusion. In other words, if the AlO*_x_* ALD overcoat is permeable for O_2_ and C_2_H_2_, the phase transition may not be prevented by the physical confinement from the AlO*_x_* ALD overcoat. Based on these observations, we believe that the lack of O_2_ and C_2_H_2_ permeabilities through the AlO*_x_* ALD overcoat, rather than the physical confinement effect, is responsible for the absence of phase transitions from Pd to PdO and PdC*_x_*.

**Figure 5:**
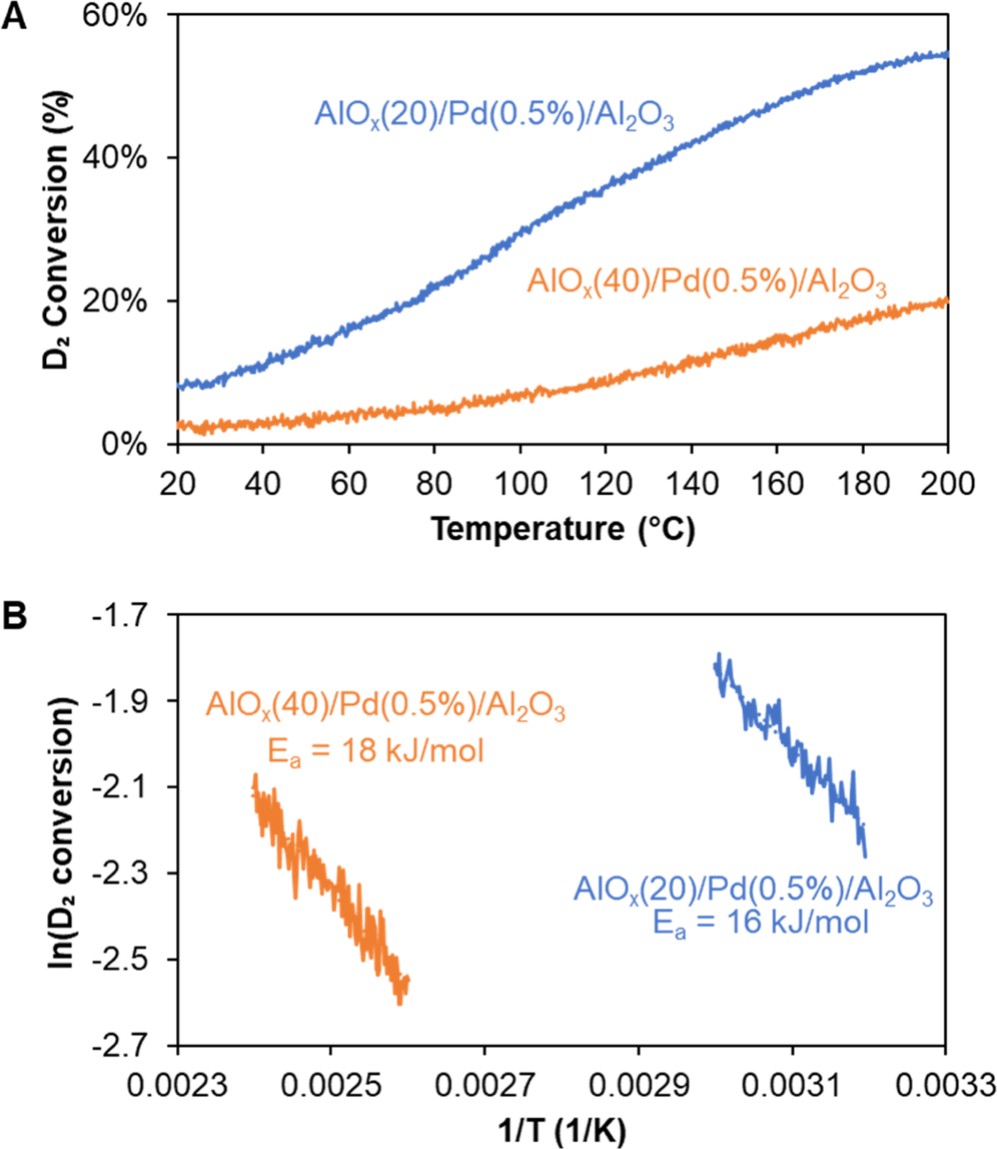
(A) D_2_ conversion on AlO*_x_*(20)/Pd(0.5%)/Al_2_O_3_ and AlO*_x_*(40)/Pd(0.5%)/Al_2_O_3_. (B) Arrhenius plots of AlO*_x_*(20)/Pd(0.5%)/Al_2_O_3_ and AlO*_x_*(40)/Pd(0.5%)/Al_2_O_3_ in the D_2_ conversion regimes of 10–16 and 8–12%, respectively.

H–D exchange studies were performed on coated Pd catalysts to investigate the accessibility of H_2_ (0.29 nm) to Pd NPs. The Pd loading was decreased to 0.5 wt % for the H–D exchange measurement because of its high activity. Figure 5A shows that the D_2_ conversion on AlO*_x_*(20)/Pd(0.5%)/Al_2_O_3_ increased from 7% at 20 °C to 54% at 200 °C, which confirms that H_2_ can access the Pd NPs through the AlO*_x_* overcoat. Increasing the ALD cycle number to 40 caused a decrease in H–D exchange activity. Furthermore, the apparent activation energies for H–D exchange on AlO*_x_*(20)/Pd(0.5%)/Al_2_O_3_ and AlO*_x_*(40)/Pd(0.5%)/Al_2_O_3_ were measured to be 16 and 18 kJ/mol, respectively (Figure 5B), whereas metallic Pd is known to be barrierless for H_2_ activation. The activation energy of H_2_ on coated Pd is close to the values reported for the heterolytic dissociation of H_2_,^46−49^ implying that the AlO*_x_* overcoat might cause H_2_ to dissociate heterolytically at the Pd–AlO*_x_* interface.

**Scheme 1:**
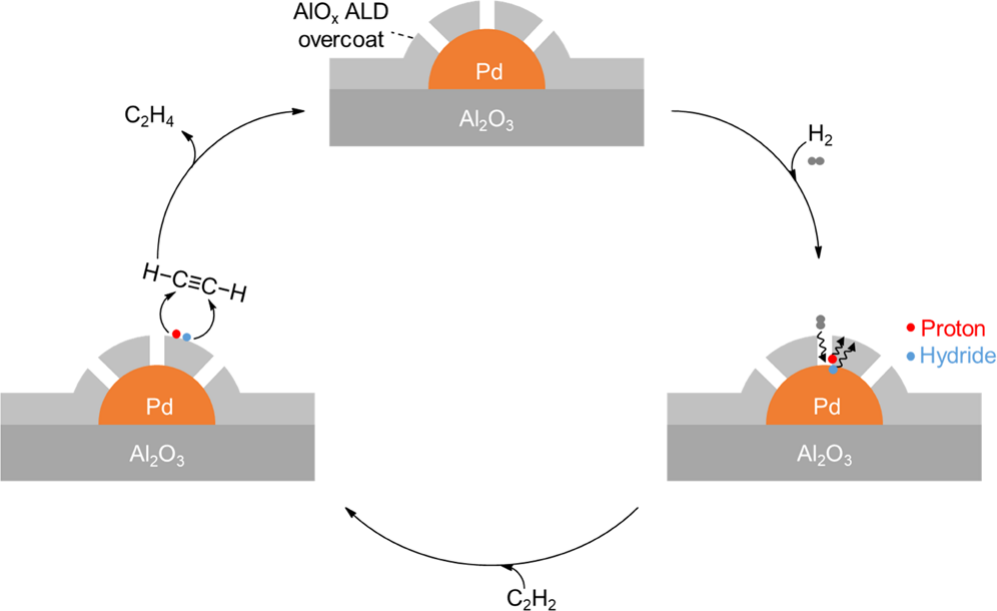
Proposed Reaction Mechanism of Acetylene Hydrogenation on Overcoated Pd Catalysts

The size-selective permeation behavior of the AlO*_x_* overcoat might originate from the amorphous nature of its pseudo-boehmite structure.^38^ Unlike crystalline materials, an amorphous ALD overcoat is full of defects, which give rise to sub-nanometer-sized internal voids. An atomic-level understanding of the amorphous structure is a challenging task and requires more research efforts.^37^ Bearing in mind that the AlO*_x_* overcoat blocks CO, O_2_, and C_2_H_2_ but allows H_2_ to access Pd NPs, we propose that C_2_H_2_ hydrogenation on overcoated Pd catalysts might undergo a hydrogen spillover mechanism: H_2_ permeates through the AlO*_x_* overcoat and dissociates heterolytically at the Pd–AlO*_x_* interface and then spills over the AlO*_x_* overcoat as a proton–hydride pair (Scheme 1), similar to that proposed by Karim et al.^32^ C_2_H_2_ is hydrogenated to C_2_H_4_ on the AlO*_x_* overcoat via proton–hydride transfer. The spillover of hydrogen from strong binding surfaces (Pd or Pt) to weak binding surfaces (Cu, Ag, or Au) has been known to suppress the overhydrogenation selectivity.^27,41,50−53^ On the other hand, the proton–hydride transfer hydrogenation might also contribute to the high selectivity toward alkenes, as reported on Ni@Chabazite,^47^ Ni/CeO_2_,^54^ Ga/CeO_2_,^55^ Ru/Al_2_O_3_,^56^ InO*_x_*,^57^ CeO_2_,^58−61^ Pd_3_S,^62^ etc. For uncoated Pd catalysts, the Pd surface is blocked by C_2_H_2_ molecules at low conversions, which inhibits the overhydrogenation of ethylene and propylene. Once C_2_H_2_ is fully converted, the Pd surface is open and gives overhydrogenation. In contrast, C_2_H_2_ cannot access and block the coated Pd surface. Therefore, the product selectivity on coated Pd is less sensitive to C_2_H_2_ conversion. This behavior is advantageous for the commercial C_2_H_2_ hydrogenation process because of its broader temperature window for operation and insensitivity to C_2_H_2_ concentration variations.

## Conclusions

In conclusion, we demonstrated that an amorphous AlO*_x_* ALD overcoat on a supported Pd catalyst could greatly suppress alkane selectivity in acetylene hydrogenation. IR spectroscopy with CO as a probe molecule showed no CO peaks on overcoated Pd, indicating that the AlO*_x_* overcoat blocks the access of the CO molecule (0.38 nm). The accessibilities of O_2_ (0.35 nm) and C_2_H_2_ (0.33 nm) to Pd NPs were probed by means of XRD based on the conversion of Pd to PdO or PdC*_x_* phases. The accessibility of H_2_ (0.29 nm) was studied using H–D exchange between H_2_ and D_2_. These studies reveal that the amorphous AlO*_x_* overcoat is able to block CO, O_2_, and C_2_H_2_ but allows the access of H_2_ to Pd surfaces. The activation energy for H–D exchange between H_2_ and D_2_ is close to the value for the heterolytic dissociation of H_2_. Therefore, we propose that H_2_ is heterolytically dissociated at the Pd–AlO*_x_* interfaces and then spills over the AlO*_x_* overcoat where acetylene hydrogenation takes place. The ALD overcoating strategy provides a powerful tool to control the accessibility of active sites in heterogeneous catalysis. It may benefit the design of catalytic architectures to control catalytic reaction pathways.

## Experimental Section

### Materials

γ-Al_2_O_3_ (NanoDur, 30–40 m^2^/g) was purchased from Alfa Aesar; SiO_2_ (fumed silica, ∼200 m^2^/g) was purchased from Sigma-Aldrich. Sodium tetrachloropalladate(II) trihydrate (Na_2_PdCl_4_·3H_2_O, 99%) and ammonium tetrachloropalladate(II) ((NH_4_)_2_PdCl_2_, 99%) were purchased from Strem Chemicals, Inc. Poly(vinylpyrrolidone) (PVP, *M*_w_ = 55 000), polyethyleneimine (PEI, *M*_w_ = 25 000 by LS, *M*_n_ = 10 000 by GPC), l-ascorbic acid (99%), ammonium chloride (NH_4_Cl, ≥99.5%), sodium borohydride (NaBH_4_, 98%), and trimethylaluminum (TMA, 97%) were purchased from Sigma-Aldrich. Acetone (99.5%) was purchased from Millipore Corporation. Deionized (DI) water obtained from an EMD Millipore Milli-DI Water Purification System was used in all experiments. Acetylene (5% in N_2_, UHP), propylene (UHP), hydrogen (5% in N_2_, UHP), deuterium (5% in N_2_, UHP), helium (UHP), hydrogen (UHP), carbon monoxide (5% in He, UHP), and N_2_ (research plus) were provided by Airgas.

### Synthesis and Adsorption of PEI-Pd NPs

The adsorption of PEI-Pd NPs was performed by antisolvent-induced adsorption.^39^ Seventy-five grams of 5 mM Na_2_PdCl_4_·3H_2_O aqueous solution was added to 3 wt % PEI aqueous solution under stirring (PEI/Pd = 0.03, mol/mol). The obtained solution was sonicated for 5 min and then left at room temperature under stirring for 4 h for complexation. A freshly prepared NaBH_4_ aqueous solution (2 wt %) was then added under stirring (NaBH_4_/Pd = 5, mol/mol). After 30 min of reduction, a dark-brown colloidal dispersion was obtained. For the adsorption of PEI-Pd NPs, 2 g of γ-Al_2_O_3_ (NanoDur) dispersed in 10 mL water was added to the as-synthesized PEI-Pd colloidal dispersion. Ninety milliliters of acetone (acetone/H_2_O = 1:1, v/v) was added under stirring. After centrifugation and washing twice with mixed solvents (acetone/H_2_O = 2:1, v/v) as well as pure acetone, the solids were dried at room temperature overnight and then calcined in air with a ramp rate of 1.5 °C/min to 400 °C and dwelled for 4 h. The nominal Pd loading was 2 wt %. This sample is denoted as Pd/Al_2_O_3_. The lower-loading Pd/Al_2_O_3_ samples (0.5, 0.25, and 0.1 wt %) were prepared with similar protocols.

### Synthesis and Adsorption of PVP-Pd NPs

The PVP-Pd cuboctahedral NPs (∼13 nm) were synthesized using the modified procedure mentioned elsewhere.^63,64^ In a typical synthesis, 9 mL of aqueous solution containing 105 mg of PVP, 60 mg of ascorbic acid, and 45 mg of ammonium chloride was heated at 100 °C under stirring for 10 min. Fifty-five milligrams of (NH_4_)_2_PdCl_4_ was dissolved in 3 mL of water and then added under stirring and maintained at 100 °C for 30 min. For adsorption of PVP-Pd NPs, 0.67 g of fumed silica dispersed in 22 mL of water was added to the as-synthesized PVP-Pd NPs. Seventeen milliliters of acetone (acetone/H_2_O = 0.5, v/v) was then added under stirring. After centrifugation, the solids were washed twice with diluted acetone (acetone/H_2_O = 1, v/v) and twice with pure acetone. After washing, the solids were dried at room temperature overnight and then calcined in air with a ramp rate of 1.5 °C/min to 400 °C and dwelled for 4 h. The nominal Pd loading was 3 wt %. This sample is denoted as Pd/SiO_2_.

### Atomic Layer Deposition (ALD)

AlO*_x_* overcoats were deposited using a bench-top viscous-flow atomic layer deposition reactor (GEMStar XT) at 150 °C by alternate exposure of trimethylaluminum (TMA) and water vapor using ultrahigh purity N_2_ as a carrier gas and purge gas, where both TMA and water bubblers were kept at room temperature. Each AlO*_x_* ALD cycle constitutes 90 s of TMA exposure followed by 90 s of water exposure, with 300 s of N_2_ purge between each exposure (90–300–90–300). Twenty cycles of AlO*_x_* ALD were performed on Pd/Al_2_O_3_ (AlO*_x_*(20)/Pd/Al_2_O_3_) and Pd/SiO_2_ (AlO*_x_*(20)/Pd/SiO_2_).

### Characterizations

#### Transmission Electron Microscopy (TEM)

For TEM analyses, a JEOL JEM-2011 TEM operated at 200 kV was used for imaging the as-synthesized catalysts. Aberration-corrected HAADF-STEM imaging was performed on a probe-corrected JEOL NEOARM operated at 80 kV. For colloidal Pd NPs, a few drops of colloidal dispersion of Pd NPs were deposited onto the Cu grids with a lacey carbon support and dried in air at room temperature. For dry powder samples, Cu grids with a lacey carbon support were dipped into the dry powders to adsorb samples by electrostatics.

#### X-ray Diffraction (XRD)

XRD measurements were performed on a PANalytical Empyrean multipurpose diffractometer with Cu Kα radiation (λ = 1.54 Å) operating at 45 kV and 40 mA. All of the measurements were carried out at room temperature with a 2θ range of 5–90° with a step of 0.02° with 100 s/step. For reduction, samples were reduced in 10% H_2_/N_2_ at 200 °C for 30 min. For carburization, samples were reduced in 10% H_2_/N_2_ at 200 °C for 30 min prior to carburization using 15 sccm of 5% C_2_H_2_/N_2_ at 200 °C for 30 min. For calcination, samples were calcined in air with a ramp rate of 1.5 °C/min to 600 °C (or 700, 800, 900 °C) and dwelled for 2 h. The 800 and 900 °C calcined samples were calcined in air with a ramp rate of 5 °C/min to 400 °C and dwelled for 4 h.

#### Diffuse Reflectance Infrared Fourier Transform Spectroscopy (DRIFTS)

DRIFTS measurements were performed on a Thermo Nicolet 6700 instrument with a Hg–Cd–Te (MCT) detector and a Praying Mantis high-temperature reaction chamber with KBr windows. The catalysts were pretreated with 100 sccm of 10% H_2_/He at 200 °C for 30 min. The CO adsorption was performed at room temperature and 100 °C. CO/Ar (5%) was introduced into the cell at a flow rate of 100 sccm. After the CO saturation, a He purge at a flow rate of 100 sccm was performed to remove the gas-phase CO from the cell. All of the spectra were recorded using 32 scans and a resolution of 4 cm^–1^.

### Catalytic Tests

Selective hydrogenation of acetylene was conducted in a fixed bed 1/4 in. quartz tube reactor. In a typical test, 20 mg of catalyst was mixed with 400 mg of quartz sand, reduced in 100 sccm of 10% H_2_/N_2_ at 200 °C for 30 min, cooled down to 20 °C in 100 sccm of 10% H_2_/N_2_, and switched to C_2_H_2_/C_3_H_6_/H_2_/N_2_ = 0.75:15:1.5:57.5 sccm; the reaction temperature was increased from 20 to 200 °C with a ramp rate of 1 °C/min. The gas flow rates were controlled by mass flow controllers (MKS Instruments). The products were analyzed by an on-line Agilent 490 microGC equipped with MS-5A (H_2_, O_2_, N_2_, CH_4_, CO), Plot U (CO_2_, C_2_H_2_, C_2_H_4_, and C_2_H_6_), and alumina (C_3+_ alkanes and C_3+_ olefins) columns. Each column is connected to a separate thermal conductivity detector. N_2_ was used as the internal standard for GC quantification.

H–D exchange between H_2_ and D_2_ was conducted in a fixed bed 1/4 in. quartz reactor tube. Briefly, 5 mg of AlO*_x_*(20)/Pd(0.5%)/Al_2_O_3_ was mixed with 100 mg of quartz sand, reduced in 10% H_2_/Ar (100 sccm) at 200 °C for 30 min. After cooling down to 20 °C, 150 sccm of 5% H_2_/N_2_ and 150 sccm of 5% D_2_/N_2_ were fed into the reactor. Mass signals of *m*/*z* = 2, 3, and 4 were monitored by an SRS QMS200 mass spectrometer. The reaction temperature was increased from 20 to 200 °C with a ramp rate of 5 °C/min.

## Supporting Information Available

Additional TEM images of Pd samples, catalysis results, and CO IR spectra (PDF)

## Abbreviations

ALD: atomic layer deposition
NPs: nanoparticles
IR: infrared
XRD: X-ray diffraction
TEM: transmission electron microscopy
BF-STEM: bright-field scanning transmission electron microscopy
HAADF-STEM: high-angle annular dark-field scanning transmission electron microscopy
PEI: polyethyleneimine
PVP: poly(vinylpyrrolidone)
