# Supplementary material for: Elucidating the Roles of Amorphous Alumina Overcoat in Palladium-Catalyzed Selective Hydrogenation
Source: ACS Appl Mater Interfaces. 2022 May 18;14(21):24290–8. doi: 10.1021/acsami.2c02132 (PMC9164194; doi:10.1021/acsami.2c02132)
Supplement: Supplementary file 1 — am2c02132_si_001.pdf [file am2c02132_si_001.pdf]

Supporting Information for

Elucidating the Roles of Amorphous Alumina Overcoat in Palladium-Catalyzed Selective Hydrogenation

Divakar R. Aireddy[1], Haoran Yu[2], David A. Cullen[2], Kunlun Ding[1].*

[1]Department of Chemical Engineering, Louisiana State University, Baton Rouge, LA 70803, USA.

[2]Center for Nanophase Materials Sciences, Oak Ridge National Laboratory, Oak Ridge, Tennessee 37831, USA.

*Email: kunlunding@lsu.edu

Figures S1 to S12

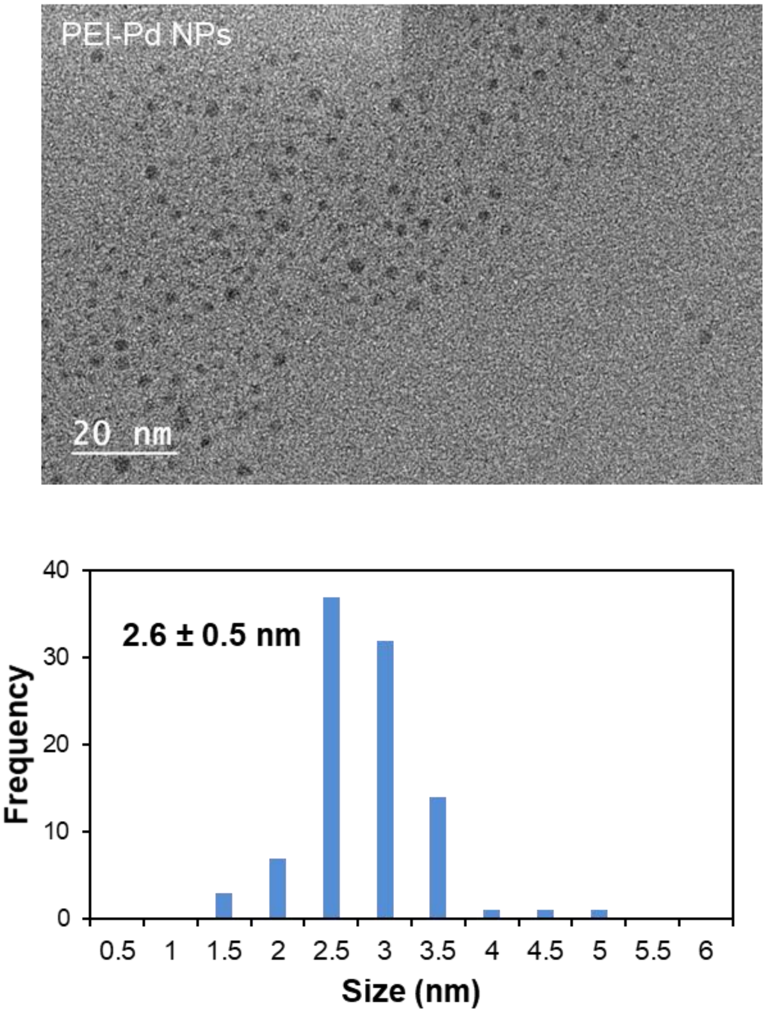

Figure S1. TEM image and size histogram of PEI-Pd NPs.

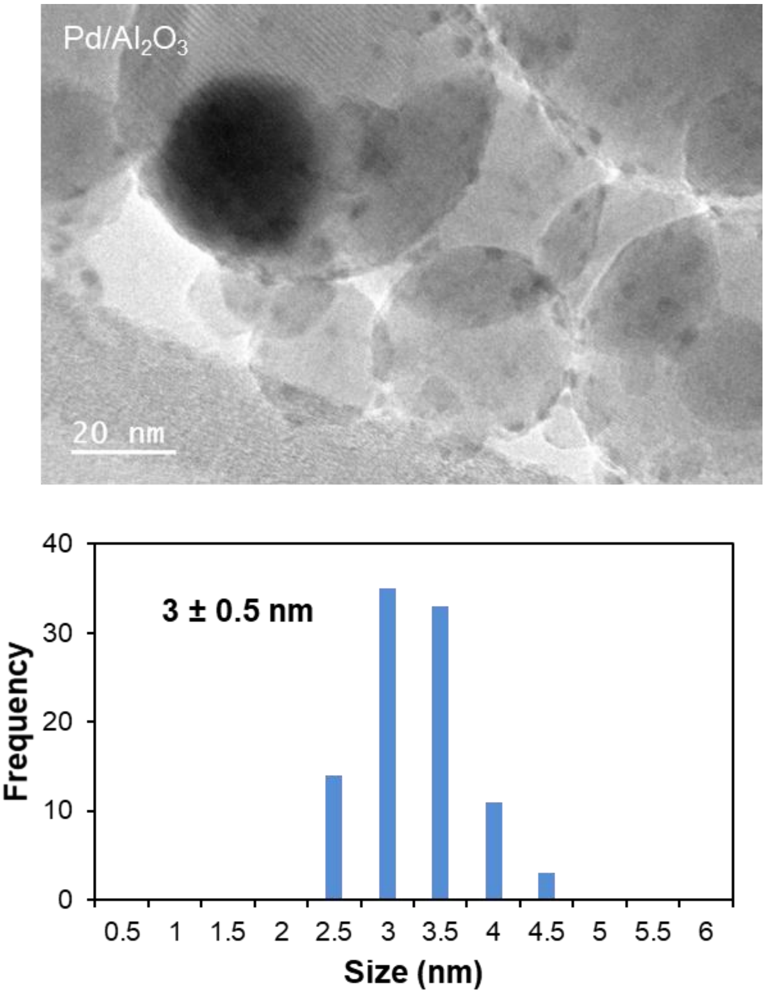

Figure S2. TEM image of Pd/Al_2O_3 and size histogram of Pd NPs.

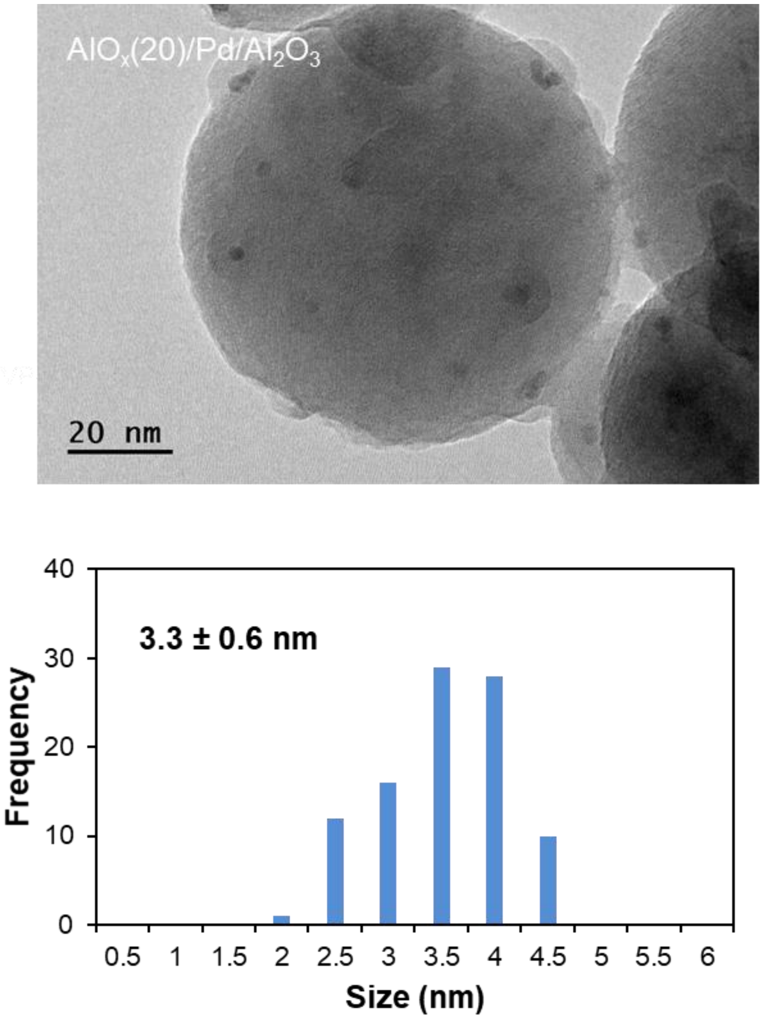

Figure S3. TEM image of $AlO_x(20)/Pd/Al_2O_3$ and size histogram of Pd NPs.

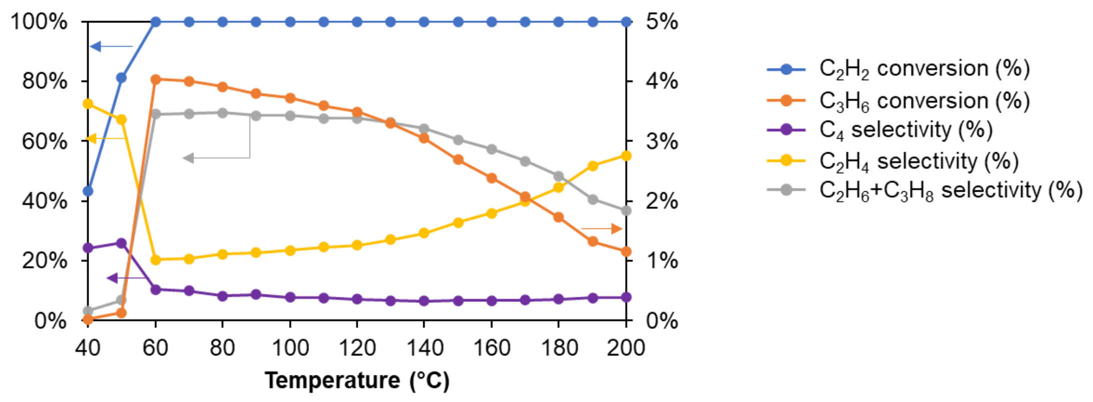

Figure S4. Conversion and product selectivity of temperature-dependent C_2H_2/C_3H_6 competitive hydrogenation over Pd/Al_2O_3.

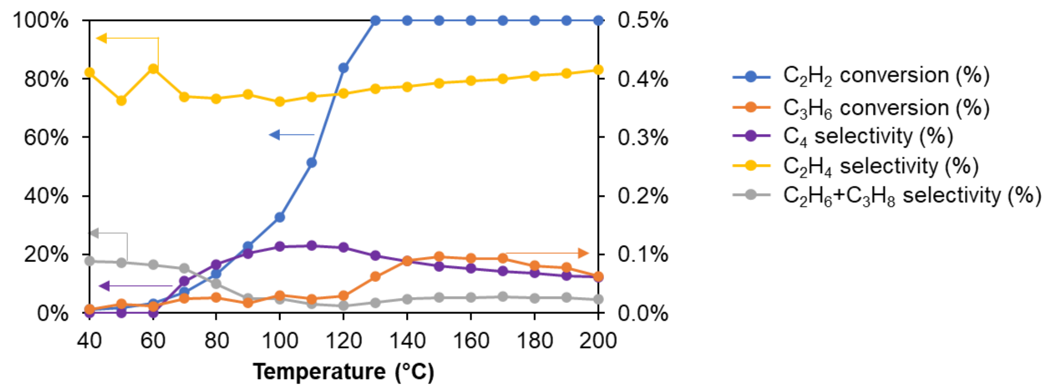

Figure S5. Conversion and product selectivity of temperature-dependent C_2H_2/C_3H_6 competitive hydrogenation over $AlO_x(20)/Pd/Al_2O_3$.

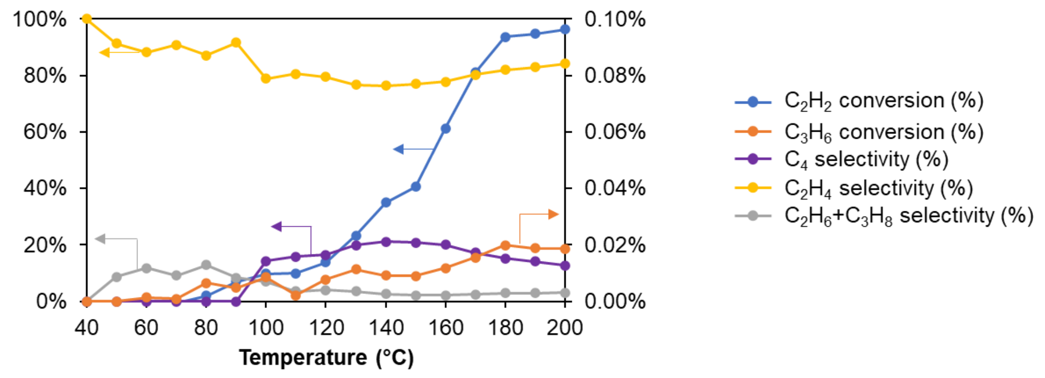

Figure S6. Conversion and product selectivity of temperature-dependent C_2H_2/C_3H_6 competitive hydrogenation over $AlO_x(40)/Pd/Al_2O_3$.

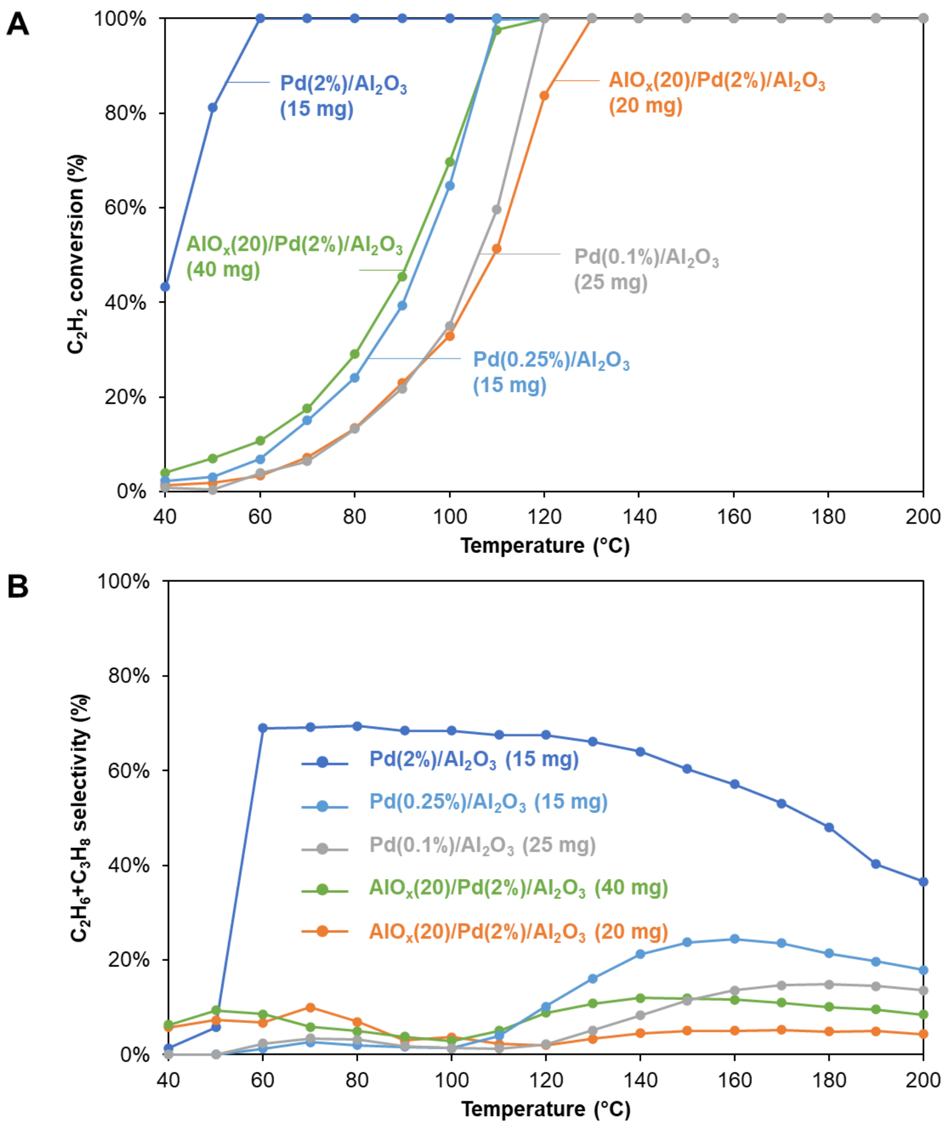

Figure S7. Comparison of catalytic performance of uncoated and overcoated Pd/Al_2O_3 in acetylene hydrogenation under similar conversions. (**A**) Acetylene conversion and (**B**) alkane selectivity as a function of temperature.

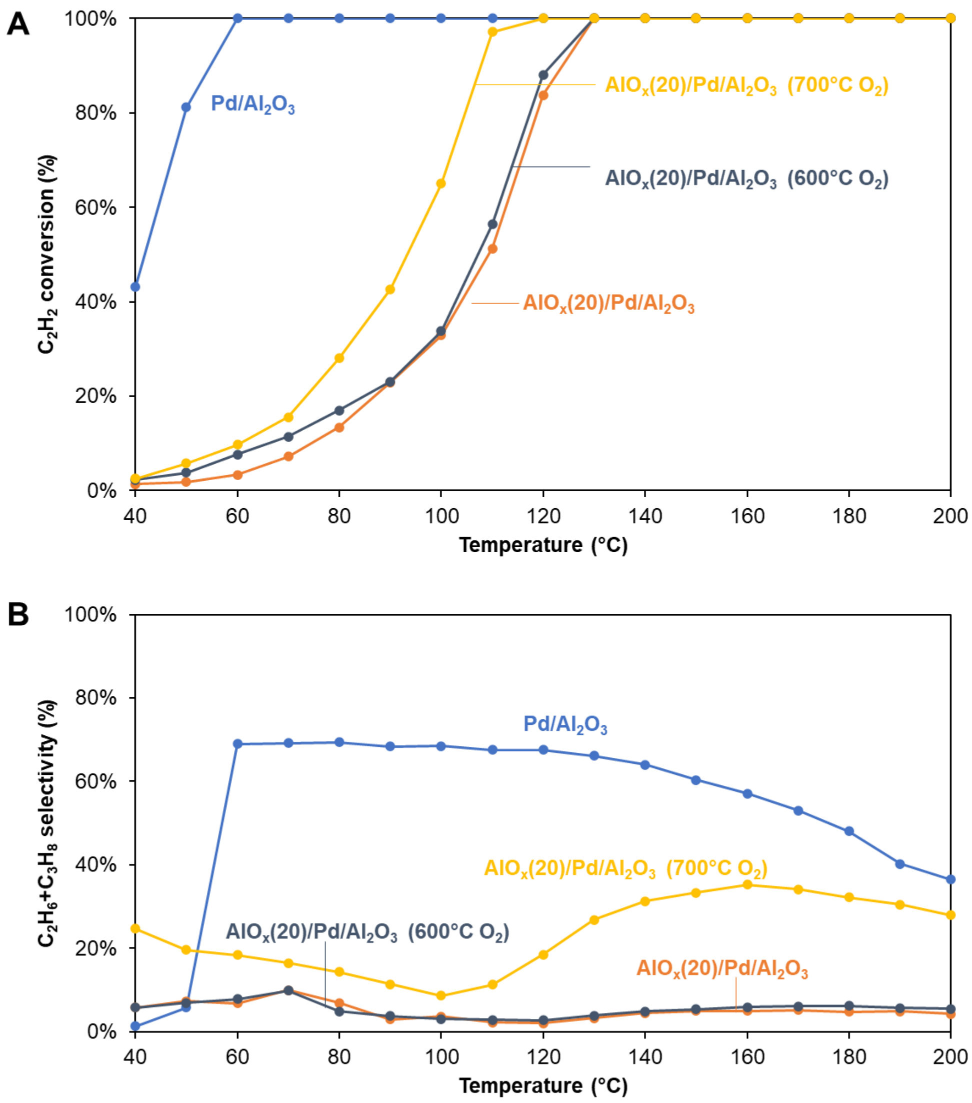

Figure S8. Catalytic performance of overcoated $AlO_x(20)/Pd/Al_2O_3$ in acetylene hydrogenation after 600°C and 700°C calcination in air. (**A**) Acetylene conversion and (**B**) alkane selectivity as a function of temperature.

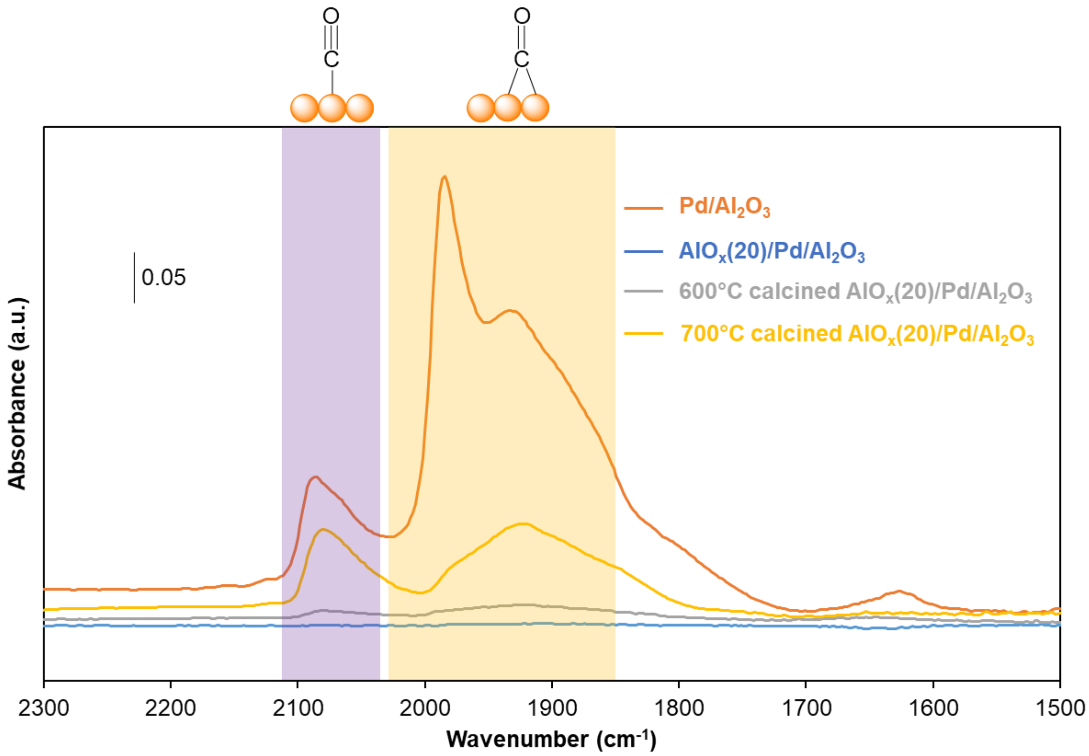

Figure S9. IR spectra of CO molecules adsorbed on uncoated and overcoated $AlO_x(20)/Pd/Al_2O_3$ after calcination at 600°C and 700°C in air.

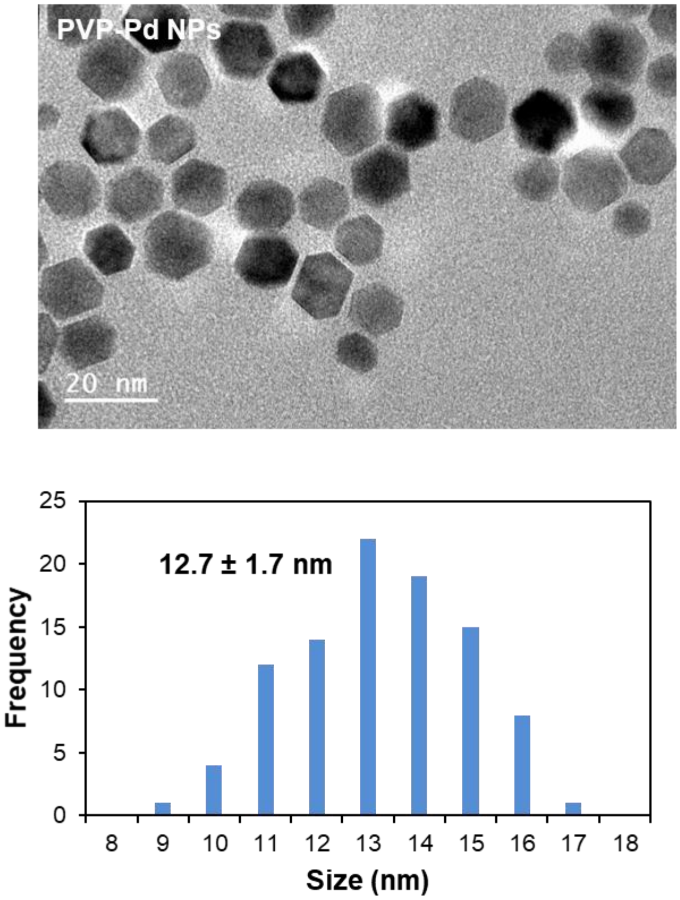

Figure S10. TEM image and size histogram of PVP-Pd NPs.

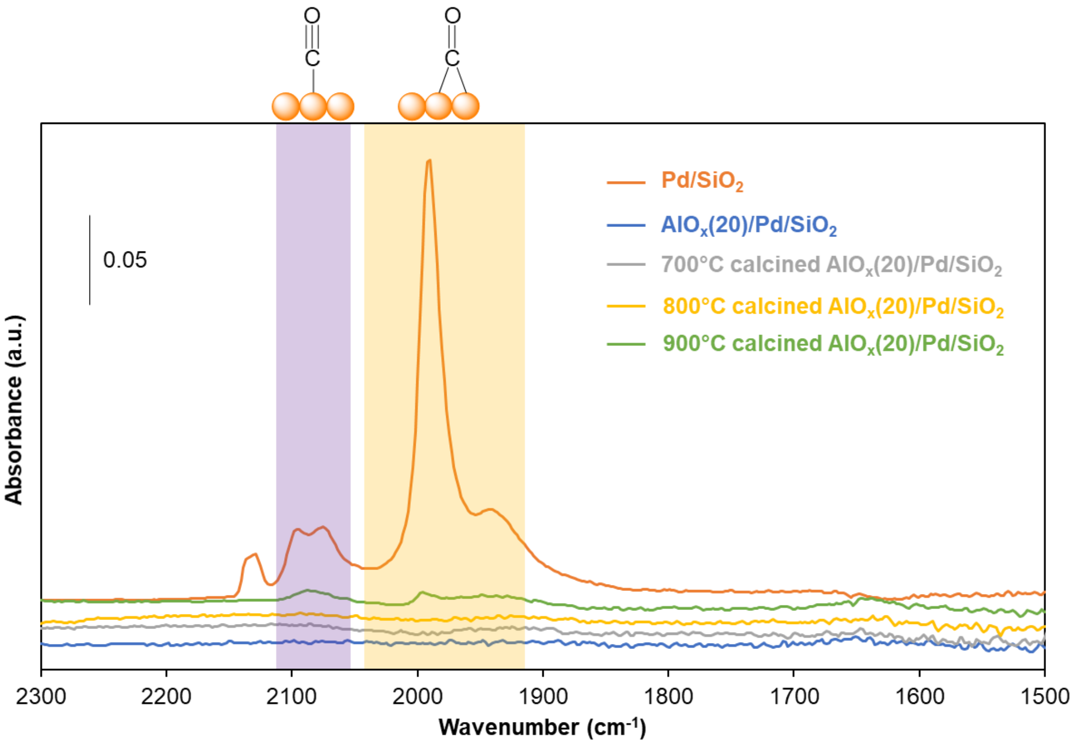

Figure S11. IR spectra of CO molecules adsorbed on uncoated Pd/SiO_2 and $AlO_x(20)/Pd/SiO_2$ at room temperature after calcination at different temperatures in air.

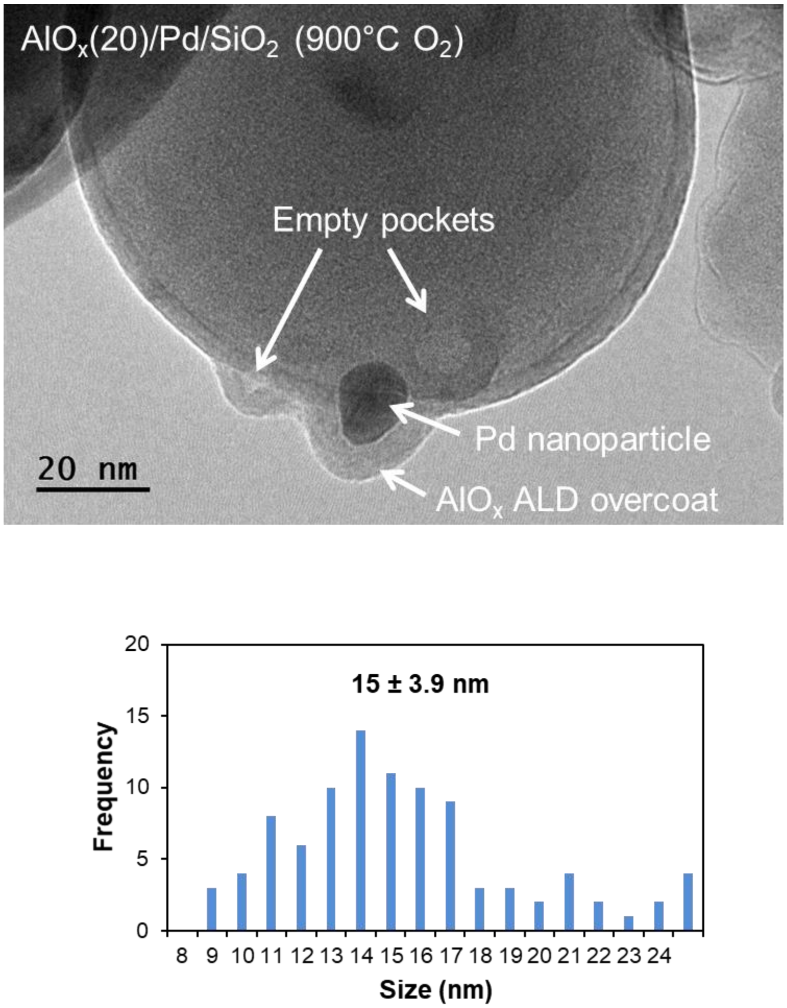

Figure S12. TEM image of $AlO_x(20)/Pd/SiO_2$ and size histogram of Pd NPs after calcination at 900°C in air.
